# An Improved Shapley Value Method for a Green Supply Chain Income Distribution Mechanism

**DOI:** 10.3390/ijerph15091976

**Published:** 2018-09-10

**Authors:** Zhongwen Xu, Zixuan Peng, Ling Yang, Xudong Chen

**Affiliations:** 1 Business School, Sichuan University, Chengdu 610064, China; xuzhongwen1996@163.com; 2 Business School, Sichuan Agricultural University, Dujiangyan 611830, China; pengzixuan99@163.com; 3 College of Management Science, Chengdu University of Technology, Chengdu 610059, China; ms_cdut@163.com

**Keywords:** environmental restoration, green supply chain, income distribution, low carbon constraints, Shapley value method

## Abstract

Low-carbon development and environmental remediation are key factors for green resource-based supply chains in China. With this aim in mind, by applying game theory under uncertain market demand, this paper incorporates low-carbon development and environmental remediation into a resource-based supply chain coordination model for decentralized and centralized markets. The results show that a centralized market can lead to improvement in total profit. Furthermore, based on an improved Shapley value method, a theoretical model for the centralized market income distribution mechanism is developed that incorporates three corrective risk factors, ecological investment, and technological level. Finally, a numerical analysis is conducted using a MATLAB simulation to obtain intuitive results, which, in turn, show the validity of incentive income distribution mechanisms for green supply chain development in China.

## 1. Introduction

Traditional resource-based supply chains are placing an increasing burden on the world [1,2,3] because they use valuable resources that emit greenhouse gases and carbon, damage ecosystems, and cause environmental problems. Resource scarcity and environmental pollution have become serious global concerns; there is now sufficient evidence to show that the current situation is no longer sustainable. To aid sustainability, in this paper, the authors develop a two-stage resource-based supply chain with two enterprises: a resource developer and a resource processor. 

Sustainability for a low-carbon economy entails low emissions, low pollution, and low energy consumption. Globally, nations have conceded that it is necessary to rapidly reduce greenhouse gas emissions, adapt to the impact of environmental degradation, and simultaneously ensure support for developing countries [4]. As the largest developing country in the world, China is under immense pressure to develop a green supply chain industry. At the Paris UN Climate Conference, China stated that, by the end of 2030, it planned to reduce its carbon intensity by 60–65% of its carbon emissions in 2005 [5]. Since then, China’s main development direction has been toward “green and low-carbon approaches”. From an enterprise perspective, resource developers perceive low-carbon activities to be associated with ecological remediation, while resource processors perceive low-carbon activities as being associated with carbon emissions reductions. Low-carbon preferences from a consumer perspective can increase the market demand for green goods that are subsidized by the government.

In this light, the Chinese government has made efforts toward sustainability. A paper titled “Implementation opinions on accelerating the construction of green mines”, was issued in 2017. It proposed specific requirements for total carbon emissions reductions and environmental remediation. Furthermore, it outlined an innovative income distribution mechanism to promote green exploration, green manufacturing, and income sharing with a view to establish a long-term mechanism for green supply chain development [6]. To respond to these government aims, we first examines the total benefit of resource-based supply chains under a low carbon constraint and different markets. Then, we discuss discusses a fair effective income distribution mechanism for resource-based supply chains. 

Our study differs from existing related studies by making the following main contributions to the current literature.

(1) We incorporate and quantify carbon emissions reductions and environmental remediation in a resource-based supply chain that includes a resource developer and a resource processor in order to determine the costs and contributions of each enterprise with regard to green development.
(2) We include ecological factors, risk factors, and other efforts to update the traditional Shapley value method in an attempt to achieve fair and effective income distribution between members, and achieve sustainable resource-based supply chain development.

The remainder of this paper is organized as follows. In Section 2, we present a comprehensive literature review; in Section 3, we outline the materials and methods, and conduct a numeric study. In Section 4, we discuss the implications of the results, and present the conclusions in Section 5.

## 2. Literature Review

Enterprises have traditionally paid greater attention to profit than environmental protection. However, as resource availability decreases, and the environment undergoes degradation, crude exploitation and utilization is no longer acceptable. As a result, there has been increased interest in recent years in sustainable supply chain development. Jeurissen et al. [7] for example, classified sustainable supply chain development based on economic, environmental, and societal perspectives, while Preuss [8] studied green supply chain design by allocating higher priority to raw materials procurement, manufacturing processes and product recovery. Corbett and Klassen [9] examined sustainable development that also protected profitability and stockholder interests. Silvestre [10] studied whether sustainability could be achieved in the oil and gas supply chain through innovation, and Walker et al. [11] summarized the unfavorable and favorable factors for green supply chain implementation. Vermeulen and Seuring [12] developed a sustainable supply chain management theory, while Klassen and Vereecke [13] examined the relationship between supply chain management, corporate social responsibility, innovation, and sustainability. Nevertheless, few of the aforementioned studies address resource-based supply chains, while most ignore low-carbon constraints imposed on enterprises, consumers’ low-carbon preference, and market demand. Therefore, protecting the environment while simultaneously achieving resource-based enterprise objectives has significant value.

With this premise, research on the relationship between carbon emissions and profits was examined. Weber and Neuhoff [14] studied carbon emissions trading and its positive impact on supply chain enterprises; Busch and Hoffmann [15] found that carbon emissions reduction could encourage green supply chain development, while Plambeck [16] analyzed the ways that companies could profitably reduce greenhouse gas emissions in their supply chains. Butner et al. [17] and Tang and Wang [18] concluded that comprehensive carbon management is closely related to sustainable supply chains, and Cholette and Venkat [19] developed software to estimate the energy usage and carbon emissions associated with supply chains and then analyzed how different supply chain configurations could impact emissions. Using the environmental Kuznets Curve, Basiri and Heydari [20] and Lau [21] studied the relationships between economic growth and carbon dioxide emissions. Finally, Chen [22] examined the relationships between the macro economy and carbon emissions input and output. On the whole, research indicates that low-carbon constraints can promote green development of resource-based supply chains. Therefore, in this paper, we examine the total benefits of the supply chain under different markets, based on this low-carbon constraint, in order to determine the most suitable one for resource-based supply chains. 

Many scholars that have sought equilibrium between green development and economic development in resource-based supply chains and individual businesses have employed game theory. For example, Patra [23] applied the Stackelberg model to study the subsidy amount, sales (retail) price and greening improvement level in a smart phone supply chain, Ren et al. [24] discussed the distribution of targets for product-related carbon emissions reductions, while Jafari et al. [25] applied the Stackelberg game-theoretic approach to find the equilibrium prices under a resource-based supply chain and provided a best-practice guide. Therefore, in this paper, before analyzing equitable income distribution, it is necessary to determine a suitable market for resource-based supply chains based on game theory.

After choosing a suitable market, we require an equitable income distribution mechanism. In other words, under the macro-control of national policies, we must guarantee incomes in each enterprise before imposing national environmental protection and low-carbon standards. If the main supply chain bodies pursue their own interests and there is unfair income distribution, this could impair the mutual trust between supply chain enterprises, and thus lower efficiency across the whole supply chain. Since few studies have examined equitable income distribution under a low-carbon constraint, seeking equilibrium between environmental protection and income distribution is vitally important. 

To ensure a fair and effective distribution of interests between all members in n-person game theory, we apply the Shapley value method. Shapley first proposed this theory in 1953. Since then, scholars have widely used it in cooperative games that distribute income based on the marginal contribution of members [26] because it assures validity, symmetry, additivity, and anonymity. However, this method has established limitations. First, it makes distribution decisions based on the marginal contributions of the members without considering the efforts made by each enterprise. Second, it is assumed that all companies bear the same risks. However, in real-world practice, factors such as risk, as well as ecological and technological levels, influence income distribution, and therefore need to be considered when using the Shapley value method.

Enterprise income is closely connected to effort and uncertain market demand. Under a low-carbon constraint, along with risk and technological (asset cost, labor cost, etc.) factors, both environmental protection and carbon emissions reductions can be incorporated when quantifying effort [27]. Low-carbon development has a positive influence on market demand [28]. As individuals become more aware of the dangers of carbon emissions and realize the importance of environmental protection, their demand for environment-friendly products increases [29,30]. Sundarakani [31], for example, concluded that people were willing to pay for environmental protection. Dawson and Segerson [32] and Laroche et al. [33] found that people were increasingly willing to pay for environment-friendly and low-carbon products, which, in turn, encouraged companies to manufacture such products. Therefore, owing to these modern-day concerns, we revise and update the traditional Shapley value method based on three factors to ensure equitable income distribution in a resource-based supply chain.

## 3. Materials and Methods 

We initially draw a genetic resource-based supply chain that can be divided into upstream and downstream members. As specific operational contents vary in their characteristics for different sectors, we specifically choose the mineral industry, which faces some of the toughest challenges of any industry in terms of low-carbon development.

**Figure 1:**
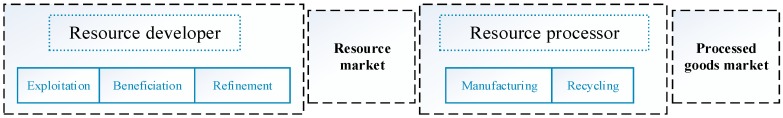
A genetic two-stage resource-based supply chain.

As shown in Figure 1, the supply chain comprises one resource developer and one resource processor. The resource developer is in charge of resource exploitation and refinement, and determine resource price. The resource processor is responsible for processed resource goods, puts them into the market, and decides on the price for the processed resource goods. We consider three decentralized decision situations: (1) the resource developer (upstream member) is in the dominant position; (2) the resource processor (downstream member) is in the dominant position; (3) the resource developer and resource processor have equal power in the market. When it comes to a centralized situation, we consider both the resource processor and developer to be an integrated unit that jointly decides prices. 

In the following sections, low-carbon and environmental remediation reflect the contributions and costs of each enterprise, low-carbon consumer preferences are incorporated to reflect market demand, and we apply game theory to analyze total profit across the whole resource-based supply chain. 

### 3.1. “Low-Carbon and Environmental Remediation” Cost to the Enterprise 

Since resource developers consider environmental pollution and destruction after resource exploitation, they tend to depict low-carbon development as ecological remediation, and the capability for which we denote using γ, and the cost for which can be written as a quadratic function of the ecological remediation capability: $C(\gamma )=\frac{1}{2}m\gamma^{2}$, $C(0)=0,C^{\prime }(\gamma )>0,C^{\prime \prime }(\gamma )>0$. As waste gas, waste water, and waste residue are produced when resources are reprocessed, resource processors view low-carbon development as emissions reduction activities. Similarly, the costs of the emissions reduction activities are related to the emissions reduction capacity, ξ, which can be written as a quadratic function of the emissions reduction: $C(\xi )=\frac{1}{2}m\xi^{2}$. In general, let *m* denote the environmental protection efforts (ecological remediation and emission reduction). The greater the *m*, the greater the cost of the input, and vice versa.

### 3.2. Market Demand that Considers the Consumers’ Low-Carbon Preferences 

Under a low-carbon constraint, consumer groups can significantly influence market demand. Consumers are willing to pay higher prices for low-carbon products in an effort to meet their “carbon behavior” of low-carbon and environmental protection preferences when consuming resource goods. Therefore, “carbon behavior” preferences have a positive influence on the market demand, ($d_{m}$), for processed goods; that is, $d_{m}=d_{0}+s\theta$, where $d_{0}$($d_{0}=\alpha -\beta p_{m}$) refers to the demand without considering environment protection, s is the coefficient for the limited “carbon behavior” preference, and θ is the environmental protection degree, and it is positively related to ecological remediation and emissions reduction. Therefore, θ can be calculated as θ=γ+ξ.

To account for market demand uncertainty from unfavorable factors, market demand is assumed to be $d_{m}=\alpha -\beta p_{m}+s\theta +\varepsilon$, where ε refers to the uncertain random terms that have a normal distribution. That is, $E(\varepsilon )=0,V(\varepsilon )=\delta_{{}_{\varepsilon }}^{2}$. 

Therefore, the market demand for processed goods is $d_{m}=\alpha -\beta p_{m}+s\theta +\varepsilon$, and the resource demand is $d_{r}=\;ad_{m}=a(\alpha -\beta p_{m}+s\theta +\varepsilon )$, where a is the resource consumption coefficient for a unit of processed goods. 

### 3.3. Decentralized Decision and Centralized Decision Situations

Current global economic conditions have resulted in increased awareness of the need for organizations to cooperate and effectively manage resources, as well as create sustainable value across the whole supply chain [34]. In the following two sections, we first examine decentralized decision and centralized decision situations to elucidate their differences. Then, we determine the most suitable market for resource-based supply chains and conduct an income distribution assessment based on the suitable market.

Under a decentralized decision situation, the resource developer and resource processor aim to pursue their own profit. Under a centralized decision situation, upstream and downstream members enter into an alliance with one another and act as a single entity. Therefore, only a single decision-maker exists to maximize the supply chain profit. Before modeling to determine the most suitable market for resource-based supply chains, we postulate the following assumptions:

(1) Upstream resource developers mainly provide resource commodities through mining and primary processing, and sell them to downstream resource processors with an optimal price; downstream resource processors mainly manufacture resource commodities provided by resource developers, and then sell to the market.
(2) Shortage and inventory costs are not considered when modeling; furthermore, resource recycling is not considered.
(3) When it comes to calculating the cost for resource developers, three costs are considered: resource exploration costs, refinement costs, and ecological restoration cost. For resource processors, costs comprise resource transportation cost, emission reduction cost, and other additional costs.

#### 3.3.1. Decentralized Decision Situation

In the decentralized market, the resource developer and processor are independent self-interested members who aim to maximize their own profits [35]. Next, we consider obtaining the equilibrium prices of the resource and processed resource goods for a supply chain. A bi-level leader-followers game model is suitable for dynamic optimization problems. That is, the Stackelberg game has the ability to fully depict the processes of the resource-based supply chain [36]. The leader is assumed to anticipate the reactions of the followers, which allows the leader to choose the best/optimal strategy accordingly [37]. In the resource-based supply chain, upstream or downstream members can act in prominent positions under decentralized decision situations. Based on Wang et al.’s work [38], which considers three possible cases, decentralized decisions should involve three game types:

(1) A downstream-dominated Stackelberg game;
(2) An upstream-dominated Stackelberg game;
(3) An upstream-and-downstream Nash game.

In the example case, the resource developer acts as the Stackelberg leader and the resource processor as the follower. Under a low-carbon constraint, we assumed that the price per resource unit from the resource developers is $p_{r}=c_{r}+c_{e}+t$ and the price set by the resource processors is $p_{m}=ap_{r}+b+\tau -\xi$. We assume that the profit function for the resource-based supply chain is a mean-variance utility function such as $\frac{1}{2}A\delta_{{}_{\varepsilon }}^{2}$, which represents the benefit fluctuations of the resource processors, where A is the risk aversion coefficient [39]. 

Therefore, the utility profit function in each single phrase is $E(\pi_{r})=E[at(\alpha -\beta p_{m}+s\theta +\varepsilon )-\frac{1}{2}m\gamma^{2}-\frac{1}{2}A{\left(a\delta_{\varepsilon }\right)}^{2}]=E[at(\alpha -\beta p_{m}+s\theta )-\frac{1}{2}m\gamma^{2}-\frac{1}{2}A{\left(a\delta_{\varepsilon }\right)}^{2}]$ and $E(\pi_{m})=E[\tau (\alpha -\beta p_{m}+s\theta )-\frac{1}{2}m\xi^{2}-\frac{1}{2}A\delta_{\varepsilon }{}^{2}]$. 

According to the Stackelberg game, the equilibrium prices are gained as follows: $p_{r(D)}^{*}=\frac{\alpha +3\beta a(c_{r}+c_{e})-\beta b+\beta \xi +s\theta }{4\beta a}$ and $p_{m(D)}^{*}=\frac{3\alpha +\beta a(c_{r}+c_{e})+\beta b-\beta \xi +3s\theta }{4\beta }$. 

The profits of each member are $E(\pi_{r(D)}^{*})=\frac{{\left[\alpha -\beta a(c_{r}+c_{e})-\beta b+\beta \xi +s\theta \right]}^{2}}{16\beta }-\frac{1}{2}m\gamma^{2}-\frac{1}{2}A{\left(a\delta_{\varepsilon }\right)}^{2}$, $E(\pi_{m(D)}^{*})=\frac{{\left[\alpha -\beta a(c_{r}+c_{e})-\beta b+\beta \xi +s\theta \right]}^{2}}{8\beta }-\frac{1}{2}m\xi^{2}-\frac{1}{2}A\delta_{\varepsilon }{}^{2}$. Then, total expected supply chain profit is equal to:

$$E(\pi_{\left(D\right)}^{*})=E(\pi_{r(D)}^{*})+E(\pi_{m(D)}^{*})=\frac{3{\left[\alpha -\beta a(c_{r}+c_{e})-\beta b+\beta \xi +s\theta \right]}^{2}}{16\beta }-\frac{1}{2}m\gamma^{2}-\frac{1}{2}m\xi^{2}-\frac{1}{2}A{\left(a\delta_{\varepsilon }\right)}^{2}-\frac{1}{2}A\delta_{\varepsilon }{}^{2}.$$

To make this paper concise and readable, another two cases under decentralized decision situation are discussed in Appendix B.

#### 3.3.2. Cooperative Centralized Decision Situation

When the resource developers cooperate with the resource processors in a resource-based supply chain, we consider them to be an integrated business unit that jointly decides equilibrium prices. They first sell the processed resource goods in the market, gain a common expected profit E(π), and distribute the income based on certain rules. Therefore, under low-carbon development, the model for E(π) is:

$$\begin{array}{l} E(\pi_{\left(T\right)})=E[(p_{m}-b+\xi )(\alpha -\beta p_{m}+s\theta )-(c_{r}+c_{e})a(\alpha -\beta p_{m}+s\theta )-\frac{1}{2}m\xi^{2}-\frac{1}{2}m\gamma^{2}-\\ \frac{1}{2}A\delta_{\varepsilon }{}^{2}-\frac{1}{2}A{\left(a\delta_{\varepsilon }\right)}^{2}] \end{array}$$

Taking the partial derivative of $E(\pi_{\left(T\right)})$ to $p_{m}$ and making $E(\pi_{\left(T\right)})$ zero, the $p_{m}$ under a maximum return is:

$$p_{m(T)}{}^{*}=\frac{\alpha +\beta a(c_{r}+c_{e})+\beta b-\beta \xi +s\theta }{2\beta }$$

Correspondingly, the expected profit of the entire resource-based supply chain is:

$$E(\pi_{\left(T\right)}^{*})=\frac{{\left[\alpha -\beta a(c_{e}+c_{r})-\beta b+\beta \xi +s\theta \right]}^{2}}{4\beta }-\frac{1}{2}m\xi^{2}-\frac{1}{2}m\gamma^{2}-\frac{1}{2}A\delta_{\varepsilon }{}^{2}-\frac{1}{2}A{\left(a\delta_{\varepsilon }\right)}^{2}$$

After the establishment and computations based on game theory in Section 3.3 and Appendix B, we obtain the anticipated maximal incomes for the resource developer and resource processor, as shown in Table A1. We conclude that, under the same emissions reduction level, the total profit under a centralized decision-making situation is greater than that under a decentralized decision-making situation. This is because the centralized decision-making condition promotes emissions reductions and ecological remediation, while a decentralized decision-making situation does not. Therefore, centralized decision-making is the best choice for resource-based enterprises.

In the next section, we discuss income distribution under a cooperative centralized decision situation.

### 3.4. The Income Distribution Mechanism

Cooperative income distribution in a resource-based supply chain should conform to the individual inputs. To achieve this, we revise and update the traditional Shapley value method based on the factors that affect income distribution in resource-based supply chains. 

#### 3.4.1. Income Distribution Principles 

In addition to individual and collective rational principles, the fair and effective income distribution in resource-based supply chains should meet the following principles:

##### Symmetry between Risk and Return

Supply chain risk refers to the uncertainty of the supply chain’s returns, which is essentially the risk result. The resource-based supply chains and the resource reserve have certain characteristics, making the supply of resource goods from the resource developers relatively stable. Thus, we can ignore the supply risks. However, given the mechanics of supply and demand, unstable market demand can result in fluctuations for resource processing enterprises, which, in turn, results in fluctuations in the market for resource goods. Under the principle of fairness and efficiency when allocating income, it is necessary to account for enterprise profits and supply chain stability. Therefore, the enterprises that bear greater risk should be allocated additional income to achieve symmetry between risk and return, and ensure stability and the continuous operation of the resource-based supply chain. In other words, the cooperative return allocated to each supply chain enterprise should be directly proportional to the risk each enterprise bears, which can be expressed mathematically as:

$$\frac{\varphi_{1}}{\delta_{1}}=\frac{\varphi_{2}}{\delta_{2}}=\cdots \frac{\varphi_{i}}{\delta_{i}}=k$$

where $\varphi_{i}$ is the return given to the i enterprise in the cooperative resource-based supply chain, and $\delta_{i}$ is the risk taken by the i enterprise in the cooperative resource-based supply chain

##### Symmetry between Low-Carbon Input and Return

As economies grow, resource and environmental problems greatly hinder economic and social development. Resource conservation and environmental protection, therefore, have become a major concern to all industry sectors. This can be either external influences arising from low-carbon constraints of the law or “green” consumer preferences, or internal requirements of enterprises to take responsibility for environmental protection. Therefore, as resource-based supply chains need to be actively engaged in environmental protection, and low-carbon activities can affect prices and returns, enterprises that have made greater efforts toward reducing environmental pollution and low-carbon development should be given a greater share of the returns. This would help in achieving symmetry between low-carbon input and returns. Thus, enterprises can be encouraged to actively protect and contribute to the environment and ecological development. This enhances green resource supply chain development. In other words, the returns given to each resource-based supply chain enterprise are proportional to their environmental protection input, which is expressed as follows:

$$\frac{\varphi_{1}}{\varsigma_{1}}=\frac{\varphi_{2}}{\varsigma_{2}}=\cdots \frac{\varphi_{i}}{\varsigma_{i}}=k^{\prime }$$

where $\varsigma_{i}$ is the environmental protection input of enterprise i in the cooperative resource-based supply chain. 

##### Symmetry between Efforts and Return

“Effort makes the difference” is a commonly accepted maxim. In terms of the resource-based supply chain income distribution, effort here mainly refers to other resources (excluding ecological factors), including tangible resources (equipment, cost of resources) and intangible resources (energy and effort needed for cooperative alliance). Therefore, income distribution should not only ensure sufficient enterprise income, but also ensure that the enterprises that make greater contributions are treated accordingly. That is, the return should be consistent with the contribution. Correspondingly, the return given to the enterprises in resource-based supply chains is proportional to their input into other resources, as follows:

$$\frac{\varphi_{1}}{\chi_{1}}=\frac{\varphi_{2}}{\chi_{2}}=\cdots \frac{\varphi_{i}}{\chi_{i}}=k^{\prime \prime }$$

where $\chi_{i}$ represents the other resources invested by *i* enterprise in the cooperative resource-based supply chain.

**Figure 2:**
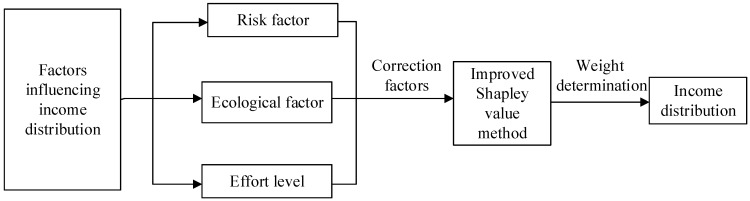
Improved Shapley value method.

As the Shapley value method distributes cooperative returns based on marginal contributions, it ignores the special participant efforts and the risk characteristics in resource-based supply chains. Therefore, based on the income distribution principles, we incorporate three correction factors to revise the Shapley value method, as shown in Figure 2.

#### 3.4.2. Quantification of Correction Factors

##### Risk Factor Quantification

The risks in resource-based supply chains include uncertain market fluctuations. Generally speaking, there is a risk return fluctuation from the resource cost input and the risk parameter. As resource-based supply chain enterprises find it difficult to identify the risk parameter of a specific resource, they tend to take the fluctuation of the benefit gained from the entire resource input. In this paper, the market fluctuations that affect the supply chain resource processor indirectly affect the benefit fluctuation. Generally, an increased risk benefit fluctuation results in a higher risk for a member enterprise, the corresponding mathematical formula for which is:

$$\frac{\delta_{1}}{R_{1}}=\frac{\delta_{2}}{R_{2}}=\cdots =\frac{\delta_{i}}{R_{i}}$$

where $R_{i}$ denotes the benefit fluctuation of the i supply chain enterprise 

We depict the risk factor based on the return after the benefit fluctuation of a member enterprise. That is, participator *i* obtains a proportional benefit of:

$$\nu_{i}=\frac{R_{i}}{\sum_{i=1}^{n}R_{i}}$$

##### Ecological Factor Quantification

We mainly study the eco-development effort level required by resource-based supply chain enterprises based on their differentiated environmental protection inputs. The cost of the effort required for ecological remediation by the resource developer and for carbon emissions reductions by resource processors is therefore consistent with the return. That is, the c participator *i* obtains is a proportional benefit of:

$${ο}_{i}=\frac{\varsigma_{i}}{\sum_{i=1}^{n}\varsigma_{i}}$$

##### Technological Level Quantification

The technological level mainly refers to the input of costs other than environmental protection, such as asset costs, labor costs, inventory costs, and warehousing costs. In this paper, the other costs input by the resource developer comprise resource development (ore selection) and resource exploration (ore prospecting), while the other costs input by the resource processors are the resource product costs associated with resource selection: processing costs, transfer costs, and other additional costs. However, we do not consider stock-out, inventory, and recycling costs. That is, participator *i* obtains a proportional benefit of:

$$\kappa_{i}=\frac{\chi_{i}}{\sum_{i=1}^{n}\chi_{i}}$$

#### 3.4.3. Improved Shapley Value Method 

As discussed, the traditional Shapley value method only considers the influence factor of each enterprise as $\frac{1}{n}$, and disregards the influence of other factors. So that the Shapley value method can be applied to the cooperative resource-based supply chain game model, we incorporate three factors into the traditional Shapley value method; the risk factor, the ecological factor, and the effort level. We introduce a comprehensive correction factor $\Delta \lambda_{i}$, and establish an improved resource-based supply chain income distribution:

$$\varphi_{i}^{\prime }=\varphi_{i}+\Delta \lambda_{i}\times V(S)$$

$$\Delta \lambda_{i}=\lambda_{i}-\frac{1}{n}$$

where $\varphi_{i}^{\prime }$ is the anticipated benefit for enterprise *i* after improvement and under a cooperative decision situation, Δλ is the difference between the comprehensive evaluation value and the average value level introduced by enterprise *i*; that is, the comprehensive correction value. Thus, $\sum_{i=1}^{n}\Delta \lambda_{i}=0$. $\lambda_{i}$ is the comprehensive evaluation value for the risk factor, the ecological factor, and the technological level of enterprise *i*, where $\sum_{i=1}^{n}\lambda_{i}=1$, V(S) is the benefit for set S. $\nu_{i},{ο}_{i},\kappa_{i}$ are the risk factor, ecological factor, and technological level input values for enterprise *i*. The computation method is illustrated in detail above, and we determine the weights of the three influence factors $w_{i}$ using AHP.

## 4. Numeric Study

### 4.1. Experimental Solution Process

We aim to examine the practicality of the proposed model in the mineral sector. This sector faces some of the toughest challenges of any industry in terms of carbon reduction and environmental remediation. The process of the mineral sector is conceptualized as a two-stage supply chain, including a resource developer and a resource processor. 

The following parameter value assumptions refer to a previously published paper [40]: α=60, $c_{r}=3$, $c_{e}=1$, b=1, a=0.8, β=5, A=0.5, m=40, τ=t=2, $\delta_{\varepsilon }=0.5$, ξ=0.8 and γ=0.4.

#### 4.1.1. Determination of Correction Factors 

Based on the risk factor, the correction factor $\nu_{m}=\frac{\frac{1}{2}A\delta_{\varepsilon }{}^{2}}{\frac{1}{2}A\delta_{\varepsilon }{}^{2}+\frac{1}{2}A{\left(a\delta_{\varepsilon }\right)}^{2}}=0.56$ is calculated for the resource processor and the corresponding $\nu_{r}=0.44$ for the resource developer. 

Based on the ecological factor, ${ο}_{m}=\frac{\frac{1}{2}m\xi^{2}}{\frac{1}{2}m\xi^{2}+\frac{1}{2}m\gamma^{2}}=0.8$ is calculated for the resource processor and ${ο}_{r}=0.2$ for the resource developer. 

Based on the other effort level in the resource-based supply chain, $\kappa_{m}=\frac{\tau (\alpha -\beta p_{m}+s\theta )}{\tau (\alpha -\beta p_{m}+s\theta )+at(\alpha -\beta p_{m}+s\theta )}=\frac{\tau }{\tau +at}=0.625$ is calculated for the resource developer and $\kappa_{r}=0.375$ for the resource processor.

#### 4.1.2. Weight Determination for the Correction Factors 

As the influences of the effort levels (risk factor, ecological factor, and technological level) are different, the enterprises in the resource-based supply chain must comprehensively consider them, with the different weights being denoted by $w_{\alpha }$, $w_{\beta }$ and $w_{\gamma }$. As combining expert judgment with the mathematical model can give more accurate results, AHP, a well-known qualitative and quantitative analysis and decision method, was deemed suitable. Thus, we consider the following steps:

- Construct the hierarchy model: the hierarchy is divided into three layers—an uppermost goal layer, a criterion layer, and a program layer (in which the index exists)
- Establish the judgment matrix: after experiments and a pairwise comparison of the significant magnitudes of two factors, Saaty and other scholars found that a nine-level ratio scale was most suitable; that is, frequently used numerical judgments (1, 3, 5, 7, 9 and 2, 4, 6, 8 there-between) that correspond to written narrative evaluations
- Hierarchical single arrangement and the corresponding consistency test: we perform a consistency test to obtain reasonable factor weights using the same test approach as hierarchical single arrangements
- Hierarchical overall arrangement and the corresponding consistency test and the specific construction of the hierarchical model

Based on the computation formulas introduced above, the qualitative evaluation standard, the collected data, the expert feedback, and the standardized index added values are shown in Table 1, from which a judgment matrix and the indicator weight levels are obtained.

MATLAB software was used to calculate the consistency index (CI) and the consistency ratio (CR). The results are $CI=\frac{\lambda_{\max}-n}{n-1}=\frac{\;3.0037-3}{3-1}=0.0018$ and $CR=\frac{CI}{RI}=\frac{0.0018}{0.58}=0.0032<0.1$, (CI and CR are the consistency text index; for specific RI, please refer to Table 2), which indicates that the matrix consistency is satisfied and the index weights are available. 

According to the weight vector $w_{i}=\left(\begin{array}{ccc} 0.1095 & 0.3090 & 0.5816 \end{array}\right)$, the technological level is given the largest weighting proportion. In other words, an enterprise’s resource input maximally influences the income distribution under a cooperative decision situation, which conforms to reality. As the resource input is a pre-condition for manufacturing and operations, and the enterprise cost affects enterprise benefit, the technological level has the highest influence weight. The resource-based supply chains must now pay attention to sustainable green development because of its strong negative externality, green production, and development requirements. Otherwise, the market will reject it. To stimulate the enterprise’s green development, the green level is the condition needed to sustain supply chain development. Therefore, the green level weight is the second most important weight. As the risk analysis indicates that the risk influence for resource-based supply chain is relatively small, the risk factor weight is the least important. From an analysis based on reality, the risk factor weight distribution conformed to the expert judgments and was therefore deemed credible.

#### 4.1.3. Determination of Correction Factors

The comprehensive correction factor, denoted by $\lambda_{i}$, is jointly determined from the influence factor weight, $w_{i}$, and the inputs (risk factor, ecological factor and technological level). Based on the resource developer inputs $\left(\begin{array}{ccc} 0.56 & 0.8 & 0.625 \end{array}\right)$, the resource processor inputs $\left(\begin{array}{ccc} 0.44 & 0.2 & 0.35 \end{array}\right)$, and the influence factor weights $\;{\left(\begin{array}{ccc} w_{\nu } & w_{ο} & w_{\kappa } \end{array}\right)}^{T}={\left(\begin{array}{ccc} 0.1095 & 0.3090 & 0.5816 \end{array}\right)}^{T}$, we find that

$$\lambda_{i}=(\begin{array}{ccc} \nu_{i} & {ο}_{i} & \kappa_{i} \end{array})\left(\begin{array}{c} w_{\nu }\\ w_{ο}\\ w_{\kappa } \end{array}\right)=\left(\begin{array}{ccc} 0.56 & 0.8 & 0.625\\ 0.44 & 0.2 & 0.375 \end{array}\right)\left(\begin{array}{c} 0.1095\\ 0.3090\\ 0.5816 \end{array}\right)=\left(\begin{array}{c} 0.6720\\ 0.3280 \end{array}\right)$$

The comprehensive correction factor, $\Delta \lambda_{i}$ is:

$$\;\Delta \lambda_{m}=\lambda_{m}-\frac{1}{n}=0.6720-0.5=0.1720$$

and

$$\;\Delta \lambda_{r}=\lambda_{r}-\frac{1}{n}=0.3280-0.5=-0.1720$$

#### 4.1.4. Income Distribution Plans

Under a decentralized decision situation, we can determine the anticipated maximal benefits for the resource developer and resource processor can be determined as $E(\pi_{r})=14.296$ and $E(\pi_{m})=9.0575$, as shown in Table 3. This conforms to the collective rationality principle according to $V\left\{r,m\right\}>E(\pi_{r})+E(\pi_{m})$ and the individual rationality principle.

Under a cooperative centralized decision situation, the resource developer can obtain a higher income of $\varphi_{r}=37.692$, which is a sum of the values at the bottom line of Table 4. $\varphi_{r}>E(\pi_{r})$ indicates that a cooperative centralized decision situation leads to greater profit than the decentralized situation. The income distributed to the resource processor is $\varphi_{m}=32.454$, as shown in Table 5, and the income distribution using the traditional Shapley value method is first calculated as $\Phi^{\prime }=(\begin{array}{cc} 37.692 & 32.454 \end{array})$.

After incorporating the correction factor $\Delta \lambda_{i}$, the results using the improved Shapley value method are as follows:

$$\varphi_{r}^{\prime }=\varphi_{r}+\Delta \lambda_{r}\times V(S)=37.692+(-0.1720)\times 70.146=37.692-12.0651=25.6269$$

and

$$\varphi_{m}^{\prime }=\varphi_{m}+\Delta \lambda_{m}\times V(S)=32.454+0.1720\times 70.146=32.454+12.0651=44.5191$$

Therefore, the improved profit allocation vector for each resource-based supply chain enterprise is: $\Phi^{\prime }=(\begin{array}{cc} 25.6269 & 44.5191 \end{array})$, which is different from the traditional Shapley value method, which is $\left(\begin{array}{cc} 37.692 & 32.454 \end{array}\right)$. By comparison, the two distribution plans conform with the individual and collective principles, guaranteeing supply chain cooperative stability. However, the improved plan better conforms to reality and achieves the best Pareto optimization. This is because it realizes income distribution optimization between each enterprise and has the highest benefit for resource-based supply chain enterprises.

### 4.2. Testing for Robustness

To demonstrate the robustness of the proposed model, another experiment was conducted, as follows. The parameters are; α=70, $c_{r}=4$, $c_{e}=2$, b=1.5, a=0.7, β=5, A=0.5, m=45, τ=t=2, $\delta_{\varepsilon }=0.6$, ξ=0.5, γ=0.6.

Under the decentralized decision situation, the anticipated benefits for the resource developer and resource processor are $E(\pi_{r})=38.451$ and $E(\pi_{m})=38.424$, and the total profit for the resource-based supply chain is $E(\pi_{{}_{B}}^{*})=E(\pi_{r})+E(\pi_{m})=76.875$. Under the centralized decision situation, the total benefit is V{r,m}=88.548, which conforms to the collective rationality principle according to $V\left\{r,m\right\}>E(\pi_{r})+E(\pi_{m})$. Using the traditional Shapley value method, the income distributions to the resource developer and the resource processor are $\varphi_{r}=44.287$, $\varphi_{m}=44.274$ and $\varphi_{r}>E(\pi_{r})$, which further confirms that the cooperative centralized decision situation resulted in a greater profit than the decentralized situation.

In the same way, the correction factors, $\lambda_{i}$, are incorporated to improve the Shapley value method. From the resource developer inputs $\left(\begin{array}{ccc} 0.67 & 0.98 & 0.59 \end{array}\right)$, the resource processor inputs $\left(\begin{array}{ccc} 0.33 & 0.02 & 0.41 \end{array}\right)$, and the correction factors’ weights $\;{\left(\begin{array}{ccc} w_{\nu } & w_{ο} & w_{\kappa } \end{array}\right)}^{T}={\left(\begin{array}{ccc} 0.1095 & 0.3090 & 0.5816 \end{array}\right)}^{T}$, we find that

$$\;\lambda_{i}=(\begin{array}{ccc} \nu_{i} & {ο}_{i} & \kappa_{i} \end{array})\left(\begin{array}{c} w_{\nu }\\ w_{ο}\\ w_{\kappa } \end{array}\right)=\left(\begin{array}{ccc} 0.67 & 0.41 & 0.59\\ 0.33 & 0.59 & 0.41 \end{array}\right)\left(\begin{array}{c} 0.1095\\ 0.3090\\ 0.5816 \end{array}\right)=\left(\begin{array}{c} 0.543\\ 0.457 \end{array}\right)$$

Then, the comprehensive correction factor $\Delta \lambda_{i}$ is

$$\Delta \lambda_{m}=\lambda_{m}-\frac{1}{n}=0.543-0.5=0.043$$

and

$$\Delta \lambda_{r}=\lambda_{r}-\frac{1}{n}=0.3280-0.5=-0.043$$

After incorporating the correction factor $\Delta \lambda_{i}$, the results using the improved Shapley value method are:

$$\varphi_{r}^{\prime }=\varphi_{r}+\Delta \lambda_{r}\times V(S)=44.287-0.043\times 88.548=40.479$$

and

$$\varphi_{m}^{\prime }=\varphi_{m}+\Delta \lambda_{m}\times V(S)=44.274+0.043\times 88.548=48.068$$

Therefore, the improved income distribution vector for each resource-based supply chain enterprise is: $\Phi^{\prime }=(\begin{array}{cc} 40.479 & 48.068 \end{array})$, which is different from $\left(\begin{array}{cc} 44.287 & 44.274 \end{array}\right)$, as solved by the traditional Shapley value method. Both results prove the effectiveness of cooperative centralized decision-making for resource-based supply chains. The improved Shapley value method promotes a fair and realistic distribution, which enhances the environmental protection.

To analyze the different influences of ecological remediation and emissions reduction efforts, the following comparison analysis was conducted. The results are shown in Table 6 and Table 7. First, as shown in Table 6, the parameter γ=0.6 was changed to γ=0.5, then V{r,m}=94.416 and the improved income distribution was $\Phi^{\prime }=(\begin{array}{cc} 40.672 & 53.744 \end{array})$. When the parameter was changed from γ=0.6 to γ=0.8, V{r,m}=76.866, the improved income distribution was $\Phi^{\prime }=(\begin{array}{cc} 37.140 & 39.726 \end{array})$. Second, as shown in Table 7, when we increased ξ=0.5 to ξ=0.6, then V{r,m}=91.788 and the improved income distribution was $\Phi^{\prime }=(\begin{array}{cc} 39.533 & 52.245 \end{array})$. When ξ=0.5 was decreased to ξ=0.4, V{r,m}=87.178, and the improved income distribution was $\Phi^{\prime }=(\begin{array}{cc} 42.731 & 44.448 \end{array})$. 

The results indicate that with an increase in the ecological remediation effort, the optimal total profit decreases. However, with an increase in the emissions reduction effort, the optimal total profit increases, which suggests that greater subsidies should be given to resource developers to encourage them to develop more low-carbon activities. The results also indicate that the income distribution mechanism proposed in this paper increases the income distributed to each participator, indirectly increases enterprise enthusiasm and effort, and achieves resource-based supply chain coordination.

### 4.3. Managerial Insights 

#### 4.3.1. Cooperative Centralized Decision-Making Is Beneficial to the Enterprises in the Resource-Based Supply Chain

In real-world practice, enterprises in an inferior position have relatively closed access to information, independent policies, and single sales and purchase channels. They bear the greater risk. For example, a 2011 survey found that the overall profits for Chinese steelmakers were lower than the overall profits of the largest three oligarch enterprises, which confirms the results in this paper. That is, cooperative centralized decision-making can assist in increasing the income of each enterprise and the total profit across the resource-based supply chain.

#### 4.3.2. The Income Distribution Mechanism Should Be Closely Related to the Participators’ Efforts

The demand for processed resource goods is closely related to price, which is largely affected by the resource developers and processors’ costs. In accordance with the income distribution principles, the effort placed on low-carbon and environmental-remediation activities should directly affect the total profit and income distribution mechanism. The results from the improved Shapley value chain reflected this realistic and fair income distribution based on the risk, ecological, and other effort factors. This could be the basis for managerial recommendations to real-world companies. For example, in China, the Hubei Sanning Mining Limited Company (Yichang, Hubei, China) is worth studying, as it aims to become the benchmark for green mining in the industry. Its operations deal with phosphate exploitation and refinement. Hubei Sanning Chemical Industry Co., Ltd. is a large chemical company that integrates coal, phosphorus, and fine chemicals. In 2006, the Hubei Sanning Mining Limited Company and Hubei Sanning Chemical Industry Co., Ltd. joined for the purpose of collaborative exploration and development of mineral resources in the Jianshui River Mining Area, Yichang. Thus, the Hubei Sanning Mining Limited Company acts as the upstream member, while Hubei Sanning Chemical Industry Co., Ltd. acts as the downstream member in the supply chain. That is, they are in a cooperative centralized market. In real-world practice, to appeal to greener and low-carbon activities, the effort placed on low-carbon and environmental-remediation activities should directly affect the total profit and income distribution mechanism. The improved Shapley value method based on the risk, ecological, and other effort factors is more suitable to distribute incomes.

Moreover, technological level has the largest influence on income distribution. This finding is worth further research. If a participator wants to increase its distributed income, it can first reduce costs associated with resource exploitation, refinement, transferring, and manufacturing. For resource-based supply chains, compulsively increasing environmental protection efforts is not suggested. Instead, different subsidy standards should be applied to different enterprises in the resource-based supply chain. 

## 5. Conclusions

As the development of a resource-based supply chain industry can seriously damage the ecological environment, environmental problems must be reduced alongside economic development. We thus considered a response to green and low-carbon development, reduced carbon emissions, and environmental remediation in cost calculations. To determine a suitable market for resource-based supply chains, we compared total profits under different markets by applying game theory. The results indicated that decisions made under a cooperative situation could maximally optimize resource-based supply chain benefit. Cooperative decision-making could effectively manage resources and create sustainable value across the whole supply chain. The resource-based supply chain income distribution mechanism for the resource developer and resource processor was investigated, for which the traditional Shapley value method was revised to include risk, ecological, and other effort level factors as correction factors to achieve fair and effective distribution. Finally, a new income distribution mechanism for resource-based supply chain cooperation was established using the improved Shapley value method. To prove the effectiveness of the proposed model, a series of numeric studies were conducted.

To achieve green economic development, low-carbon emissions, and environmental remediation, consumers’ “low-carbon” preference behavior should be incorporated in the supply chain income distribution and coordination mechanism. This could resolve irrationalities and unfairness in income distribution in resource-based supply node enterprises, guarantee the enterprises’ maximum interest, and enhance the operational efficiency of the supply chain. Rather than compulsively increasing environmental protection efforts, different subsidy standards should be applied to different enterprises in the resource-based supply chain.

However, we only considered a two-stage resource-based supply chain that included a single developer and a single processor, while a multiple-stage supply chain may be possible. To achieve sustainable supply chain development, studying the total resource supply chain profits from one single period is insufficient—a prospective study spanning multiple periods would be valuable.

## Figures and Tables

**Table 1:** Expert judgments and weights.

Factors	Risk Factor	Ecological Factor	Technological Level	$\mathrm{Weight}\;w_{i}\;$
Risk factor	1	1/3	1/5	0.1095
Ecological factor	3	1	1/2	0.3090
Technological level	5	2	1	0.5816

**Table 2:** Average random consistency index (RI).

Matrix order (*n*)	1	2	3	4	5	6	7	8	9
RI	0.00	0.00	0.58	0.90	1.12	1.24	1.32	1.41	1.45

**Table 3:** Income under different markets.

S	$E(\pi_{r})\;$	$E(\pi_{m})\;$	V{r,m}
V(S)	14.296	9.0575	70.146

**Table 4:** Income distribution for resource developers under a cooperative centralized decision.

S	V{r}	V{r,m}
V(S)	14.296	70.146
V(S\r)	0	9.0575
V(S)−V(S\r)	14.296	61.0885
|S|	1	2
W(|S|)	0.5	0.5
W(|S|)(V(S)−V(S\r))	7.148	30.544

V(S): total benefit of set *S*; V(S\i): total benefit of set S minus participator *i*; W(|S|): weighting factor, representing the contribution of each participator in set *S*.

**Table 5:** Income distribution for the resource processor under a cooperative centralized decision.

S	V{m}	V{r,m}
V(S)	9.0575	70.146
V(S\m)	0	14.296
V(S)−V(S\r)	9.0575	55.850
|S|	1	2
W(|S|)	0.5	0.5
W(|S|)(V(S)−V(S\r))	4.529	27.925

**Table 6:** Income distribution vector under different ecological remediation efforts γ.

Ecological Remediation Effort	V{r,m}	Improved Income Distribution Vector
γ=0.5	94.416	$\Phi^{\prime }=(\begin{array}{cc} 40.672 & 53.744 \end{array})$
γ=0.6	88.584	$\Phi^{\prime }=(\begin{array}{cc} 40.479 & 48.068 \end{array})$
γ=0.8	76.866	$\Phi^{\prime }=(\begin{array}{cc} 37.140 & 39.726 \end{array})$

**Table 7:** Income distribution vector under different emission reduction efforts ξ.

Ecological Remediation Effort	V{r,m}	Improved Income Distribution Vector
ξ=0.4	87.178	$\Phi^{\prime }=(\begin{array}{cc} 42.731 & 44.448 \end{array})$
ξ=0.5	88.584	$\Phi^{\prime }=(\begin{array}{cc} 40.479 & 48.068 \end{array})$
ξ=0.6	91.788	$\Phi^{\prime }=(\begin{array}{cc} 39.533 & 52.245 \end{array})$

## Appendix A

Description of symbols:

$p_{m}$	price of a processed resource good unit
$p_{r}$	price of a resource good unit
$c_{r}$	development costs for a resource goods unit
$c_{e}$	cost of exploration for the allocation of a resource goods unit
b	additional cost of processed resource goods minus the cost of transfer and emissions reduction in the production process
t	profit of a product unit by resource developers
τ	profit of a product unit by resource processors
U	upstream dominance
D	downstream dominance
B	upstream-and-downstream equilibrium
T	cooperative centralized decision
E(π)	profit utility function for a resource-based supply chain system as well as the expected return in the paper. This paper assumes $E(\pi_{i}{}^{*})=E(\pi_{r(i)}^{*})+E(\pi_{m(i)}^{*}),i=U,D,B,T$
R.I.	mean random consistency index
CI and CR	consistency text index
$\varphi_{i}$	return given to *i* enterprise in a cooperative resource-based supply chain
$\delta_{i}$	risk taken by *i* enterprise in the cooperative resource-based supply chain
V(S)	total benefit of set *S*
V(S\i)	total benefit of set S minus participator *i*
W(|S|)	weighting factor, representing the contribution of each participator in set *S*

## Appendix B

### Appendix B.1. Upstream-Dominated Stackelberg Game

The Stackelberg game is divided into two phases when the resource developers are in the dominant position. First, the resource processors seek to maximize their expected profit based on the price nominated by the resource developers, after which they decide on the price for the processed resource goods. Then, to maximize the resource developer profit utility function, the resource developers use an optimal processed resource goods price to determine the price of the resource goods. Then the results are shown in Equations (A1)–(A5) referring to Appendix B.

(A1) $$p_{r(U)}^{*}=\frac{\alpha +\beta a(c_{r}+c_{e})-\beta b+\beta \xi +s\theta }{2\beta a}\;$$

(A2) $$p_{m(U)}^{*}=\frac{3\alpha +\beta a(c_{r}+c_{e})+\beta b-\beta \xi +s\theta }{4\beta }\;$$

(A3) $$E(\pi_{r(U)}^{*})=\frac{{\left[\alpha -\beta a(c_{r}+c_{e})-\beta b+\beta \xi +s\theta \right]}^{2}}{8\beta }-\frac{1}{2}m\gamma^{2}-\frac{1}{2}A{\left(a\delta_{\varepsilon }\right)}^{2}\;$$

(A4) $$E(\pi_{m(U)}^{*})=\frac{{\left[\alpha -\beta a(c_{r}+c_{e})-\beta b+\beta \xi +s\theta \right]}^{2}}{16\beta }-\frac{1}{2}m\xi^{2}-\frac{1}{2}A\delta_{\varepsilon }{}^{2}\;$$

(A5) $$\begin{array}{l} E(\pi_{\left(U\right)}^{*})=E(\pi_{r(U)}^{*})+E(\pi_{m(U)}^{*})\\ =\frac{3{\left[\alpha -\beta a(c_{r}+c_{e})-\beta b+\beta \xi +s\theta \right]}^{2}}{16\beta }-\frac{1}{2}m\xi^{2}-\frac{1}{2}m\gamma^{2}-\frac{1}{2}A\delta_{\varepsilon }{}^{2}-\frac{1}{2}A{\left(a\delta_{\varepsilon }\right)}^{2} \end{array}$$

### Appendix B.2. Upstream-and-Downstream Nash Equilibrium

When resource developers and resource processors have equal power, and thus play a vertical Nash game [38], then we calculate the total expected supply chain profit, as shown in Equation (A6) in Appendix B.

(A6) $$\begin{array}{l} E(\pi_{\left(B\right)}^{*})=E(\pi_{r(B)}^{*})+E(\pi_{m(B)}^{*})\\ =\frac{2{\left[\alpha -\beta a(c_{r}+c_{e})-\beta b+\beta \xi +s\theta \right]}^{2}}{9\beta }-\frac{1}{2}m\xi^{2}-\frac{1}{2}m\gamma^{2}-\frac{1}{2}A\delta_{\varepsilon }{}^{2}-\frac{1}{2}A{\left(a\delta_{\varepsilon }\right)}^{2} \end{array}$$

Table A1 shows the attainable incomes for each partner in the resource-based supply chain under differentiated markets.

**Table A1:** Equilibrium and incomes for the resource-based supply chain under differentiated markets.

Participators	Resource Developer	Resource Processor	Total Resource-Based Supply Chain Benefit
Downstream leading market	$\begin{array}{l} \frac{{\left[\alpha -\beta a(c_{r}+c_{e})-\beta b+\beta \xi +s\theta \right]}^{2}}{16\beta }\\ -\frac{1}{2}m\gamma^{2}-\frac{1}{2}A{\left(a\delta_{\varepsilon }\right)}^{2}{}_{} \end{array}$	$\begin{array}{l} \frac{{\left[\alpha -\beta a(c_{r}+c_{e})-\beta b+\beta \xi +s\theta \right]}^{2}}{8\beta }\\ -\frac{1}{2}m\xi^{2}-\frac{1}{2}A\delta_{\varepsilon }{}^{2} \end{array}$	$\begin{array}{l} \frac{3{\left[\alpha -\beta a(c_{r}+c_{e})-\beta b+\beta \xi +s\theta \right]}^{2}}{16\beta }-\frac{1}{2}m\gamma^{2}-\frac{1}{2}m\xi^{2}\\ -\frac{1}{2}A{\left(a\delta_{\varepsilon }\right)}^{2}-\frac{1}{2}A\delta_{\varepsilon }{}^{2} \end{array}$
Upstream leading market	$\begin{array}{l} \frac{{\left[\alpha -\beta a(c_{r}+c_{e})-\beta b+\beta \xi +s\theta \right]}^{2}}{8\beta }\\ -\frac{1}{2}m\gamma^{2}-\frac{1}{2}A{\left(a\delta_{\varepsilon }\right)}^{2} \end{array}$	$\begin{array}{l} \frac{{\left[\alpha -\beta a(c_{r}+c_{e})-\beta b+\beta \xi +s\theta \right]}^{2}}{16\beta }\\ -\frac{1}{2}m\xi^{2}-\frac{1}{2}A\delta_{\varepsilon }{}^{2} \end{array}$	$\begin{array}{l} \frac{3{\left[\alpha -\beta a(c_{r}+c_{e})-\beta b+\beta \xi +s\theta \right]}^{2}}{16\beta }-\frac{1}{2}m\xi^{2}-\frac{1}{2}m\gamma^{2}\\ -\frac{1}{2}A\delta_{\varepsilon }{}^{2}-\frac{1}{2}A{\left(a\delta_{\varepsilon }\right)}^{2} \end{array}$
Equilibrium market	$\begin{array}{l} \frac{{\left[\alpha -\beta a(c_{r}+c_{e})-\beta b+\beta \xi +s\theta \right]}^{2}}{9\beta }\\ -\frac{1}{2}m\gamma^{2}-\frac{1}{2}A{\left(a\delta_{\varepsilon }\right)}^{2} \end{array}$	$\begin{array}{l} \frac{{\left[\alpha -\beta a(c_{r}+c_{e})-\beta b+\beta \xi +s\theta \right]}^{2}}{9\beta }\\ -\frac{1}{2}m\xi^{2}-\frac{1}{2}A\delta_{\varepsilon }{}^{2} \end{array}$	$\begin{array}{l} \frac{2{\left[\alpha -\beta a(c_{r}+c_{e})-\beta b+\beta \xi +s\theta \right]}^{2}}{9\beta }-\frac{1}{2}m\xi^{2}-\frac{1}{2}m\gamma^{2}\\ -\frac{1}{2}A\delta_{\varepsilon }{}^{2}-\frac{1}{2}A{\left(a\delta_{\varepsilon }\right)}^{2} \end{array}$
Centralized decision market			$\begin{array}{l} \frac{{\left[\alpha -\beta a(c_{e}+c_{r})-\beta b+\beta \xi +s\theta \right]}^{2}}{4\beta }-\frac{1}{2}m\xi^{2}-\frac{1}{2}m\gamma^{2}\\ -\frac{1}{2}A\delta_{\varepsilon }{}^{2}-\frac{1}{2}A{\left(a\delta_{\varepsilon }\right)}^{2} \end{array}$
